# Clinical and Histopathological Prognostic Factors in Chondrosarcomas

**DOI:** 10.1080/13577149778470

**Published:** 1997-03

**Authors:** Søren Daugaard, Olaf Myhre-Jensen, Torben Schiødt, Anne G. Jurik, Johnny Keller, Henning T. Mouridsen, Bjarne Lund

**Affiliations:** 1 Department of Pathology 5443 Rigshospitalet (University Hospital) Frederik V's Vej 11 Copenhagen Ø DK-2100 Denmark; 2 Department of Pathology Aarhus University Hospital Denmark; 3 Department of Radiology Aarhus University Hospital Denmark; 4 Department of Orthopaedic Surgery Aarhus University Hospital Denmark; 5 Department of Oncology Rigshospitalet (University Hospital) Copenhagen Denmark; 6 Department of Orthopaedic Surgery Rigshospitalet (University Hospital) Copenhagen Denmark

## Abstract

*Purpose*. In an attempt to identify clinical and histopathological factors of prognostic importance in chondrosarcomas, 115 cases of malignant and borderline chondromatous tumours were reviewed.

*Patients/methods*. Histopathological features tested for prognostic information as well as reproducibility included cellularity, nuclear pleomorphism, multinucleated cells, mitotic activity and grade. Eleven patients had a biopsy only, and a short survival (median 2.0 years); these were excluded from further analysis. The remaining 104 patients who had received intended curative treatment had a median survival of 14.7 years.

*Results*. In univariate analysis, tumour size, extra-compartmental growth, surgical margin and sex were significantly correlated to recurrence-free survival (RFS); sex was marginally significant while age, site and pathological parameters were not significant. Overall survival (OAS) was likewise found to be independent of pathological features as well as site, size and surgical margin; but age, sex and extra-compartmental growth were statistically significant. However, when the same parameters were entered into a stepwise Cox (multivariate) analysis, only surgical margin, cellularity and pleomorphism were significantly related to RFS; margin, grade, pleomorphism and age to OAS. Overall inter-observer agreement on grade was relatively low: 0.54, with a Kappa value of 0.32. It was not better for the other histological parameters, with the exception of the mitotic count. However, acceptable values were achieved when the material was divided into low-grade (grade I and below) vs high-grade (grade II and III) lesions: overall agreement 0.79, Kappa 0.56.

*Discussion*. Although the grading of chondrosarcomas is in need of improvement, its replacement by semiquantitative evaluation of individual histopathological parameters as performed in this study offers no advantage. Among the clinical parameters, only the adequacy of the surgical treatment and the patient's age appear to be important.


*Sarcoma* (1997) **1**, 47–54

ORIGINAL ARTICLE

### Clinical and histopathological prognostic factors in chondrosarcomas

SØREN DAUGAARD,[1] OLAF MYHRE-JENSEN,[2] TORBEN SCHIØDT,[1] ANNE G. JURIK,[3] JOHNNY KELLER,[4] HENNING T. MOURIDSEN[5] & BJARNE LUND[6]

*Departments of [1]Pathology, [5]Oncology and [6]Orthopaedic Surgery, Rigshospitalet (University Hospital), Copenhagen and Departments of [2]Pathology, [3]Radiology and [4]Orthopaedic Surgery, Aarhus University Hospital, Denmark*

**Abstract**

*Purpose*. In an attempt to identify clinical and histopathological factors of prognostic importance in chondrosarcomas, 115 cases of malignant and borderline chondromatous tumours were reviewed.

*Patients/methods*. Histopathological features tested for prognostic information as well as reproducibility included cellularity, nuclear pleomorphism, multinucleated cells, mitotic activity and grade. Eleven patients had a biopsy only, and a short survival (median 2.0 years); these were excluded from further analysis. The remaining 104 patients who had received intended curative treatment had a median survival of 14.7 years.

*Results*. In univariate analysis, tumour size, extra-compartmental growth, surgical margin and sex were significantly correlated to recurrence-free survival (RFS); sex was marginally significant while age, site and pathological parameters were not significant. Overall survival (OAS) was likewise found to be independent of pathological features as well as site, size and surgical margin; but age, sex and extra-compartmental growth were statistically significant. However, when the same parameters were entered into a stepwise Cox (multivariate) analysis, only surgical margin, cellularity and pleomorphism were significantly related to RFS; margin, grade, pleomorphism and age to OAS. Overall inter-observer agreement on grade was relatively low: 0.54, with a Kappa value of 0.32. It was not better for the other histological parameters, with the exception of the mitotic count. However, acceptable values were achieved when the material was divided into low-grade (grade I and below) vs high-grade (grade II and III) lesions: overall agreement 0.79, Kappa 0.56.

*Discussion*. Although the grading of chondrosarcomas is in need of improvement, its replacement by semiquantitative evaluation of individual histopathological parameters as performed in this study offers no advantage. Among the clinical parameters, only the adequacy of the surgical treatment and the patient's age appear to be important.

**Key words**: *chondrosarcoma, prognosis, clinical parameters, histopathological parameters, grade, inter-observer variation.*

### Introduction

Chondrosarcomas follow osteosarcomas as the second most common primary malignancy of bone. Unlike the latter, there has been no real advances in the treatment of chondrosarcomas through the twentieth century. The disease is remarkably resistant to both radiotherapy and chemotherapy, and surgery is still the mainstay of the treatment.[1]

Since chondrosarcomas can vary in behaviour between extremely low grade, slowly growing expansive lesions and highly aggressive, invasive and metastasizing tumours, the surgeon has to balance between the necessity to achieve an adequate surgical margin and the wish to avoid an unnecessary and possibly disabling operation. While clinical and radiological findings are helpful when judging the aggressiveness of the tumour, the histological grade is often the major factor in determining the surgical treatment. Customarily, three-grade systems are used, with grade I indicating a slowly growing, expansive lesion and grade III signifying a locally aggressive tumour with a high risk of recurrence and metastasis.[2–4]

However, the grading of chondrosarcomas is assumed to be highly subjective and difficult even for pathologists with experience in bone pathology.[5] This is due partly to the rarity of these tumours, and partly to a weakness in the grading systems themselves. Apart from subtle differences between them, they are also mainly descriptive, i.e. they describe the typical grade I, II and III lesions but do not define clear boundaries between them, nor do they explain how the individual features should be weighted.[6,7] While this may not be a problem within individual institutions—where grading is performed by a single pathologist—it does make comparison of treatment results between the centres unreliable.

Correspondence to: S. Daugaard, Department of Pathology 5443, Rigshospitalet, Frederik V's Vej 11, DK-2100 Copenhagen Ø, Denmark. Tel: +45 35 45 53 40; Fax: +45 35 45 63 80; E-mail: sarcpath@rh.dk.

1357-714X/97/010047-08 ©1997 Journals Oxford Ltd

**Table 1.** *Histological parameters (as stratified in the survival analysis)*

| Parameter | Points | Definition |
|---|---|---|
| Cellularity* | 1 | $< 300$ cells/mm$^2$ |
| | 2 | 300–600 cells/mm$^2$ |
| | 3 | 600–900 cells/mm$^2$ |
| | 4 | $> 900$ cells/mm$^2$ |
| Nuclear pleomorphism** | 1 | Slight: uniform round/oval dark nuclei without prominating nucleoli |
| | 2 | Moderate: dark, with irregular contours or light, round/oval with distinct nucleoli |
| | 3 | Severe: bizarre giant nuclei present |
| Multinucleated cells | 1 | $< 10$/mm$^2$ |
| | 2 | 10–27/mm$^2$ |
| | 3 | $> 27$/mm$^2$ |
| Mitoses*** | 0 | None |
| | 1 | $\leq 1$/mm$^2$ |
| | 2 | $> 1$/mm$^2$ |

*Extrapolated from the maximum cell density in 0.06 mm$^2$.
**Some compensation allowed for poor fixation.
***Extrapolated from the count in at least 10 high power fields.

We therefore decided to test individual histopathological parameters for reproducibility as well as clinical relevance, together with clinical factors of known or purported prognostic importance.

### Patients and methods

### *Patients*

The material consists of 115 patients treated for histologically confirmed chondrosarcomas at the Soft Tissue and Bone Tumour Centres in Copenhagen and Aarhus in the period 1965–1994. Excluded were extraskeletal, mesenchymal and so-called dedifferentiated chondrosarcomas. Included were a few cases where the original pathology report had given a diagnosis of borderline chondromatous tumour ('possible chondrosarcoma', etc.). The following clinical parameters were selected for analysis: age, sex, tumour site and size, and surgical margin. Margin was classified according to Enneking.[8] The presence or absence of extracompartmental extension was evaluated on the combined evidence of clinical findings, radiology and pathology. Survival was calculated from the day of admission or—in the case of recurring tumours or patients referred for other reasons—from the date of the first biopsy indicating malignancy. End-points were clinically or radiologically detected local or distant recurrence (recurrence-free survival, RFS), or death (overall survival, OAS).

### *Histological analysis*

From each tumour, one or two H&E-stained slides were selected, containing the areas that were judged to be best preserved and diagnostic. These slides were then circulated between three pathologists (SD, OMJ and TS) and conventional grading was performed following the guidelines of Gitelis *et al.*,[3] with knowledge of the clinical data (age, site, size). In a second round, four individual histological parameters (cellularity, nuclear pleomorphism, multinucleated cells and mitotic count) were graded and scored according to the criteria in Table 1. Area measurements were performed with the help of ocular counting grids so that the counts could be recalculated per mm$^2$; calibration was done individually, since the optical properties of the microscopes differed. The parameters had been selected after a preliminary analysis which included calcification, myxoid changes, cytoplasmic inclusions and growth pattern; these were dropped from further analysis because they gave no prognostic information in uni- or multivariate analysis (data not shown). Invasive growth (defined as demonstrable invasion in bone or soft tissue) had shown borderline significance and was included in the present analysis, although only as evaluated by one pathologist. For the prognostic analysis, a consensus grade was agreed upon in all cases with discrepancies; in the individual parameters, the mean value was used.

### *Statistical analysis*

Only patients treated with curative intent ($n = 104$) were included in the survival analysis. First, a univariate analysis was performed by calculating RFS and OAS with log-rank tests for each of the stratified variables. Next, the same variables were subjected to a multivariate analysis using Cox's proportional hazards model in a stepwise fashion, with

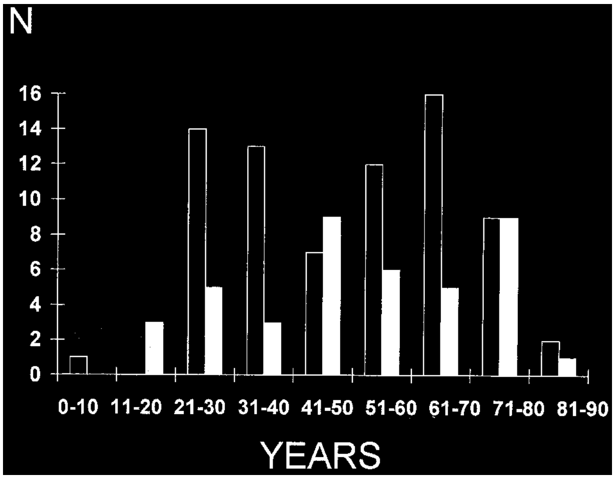

**Fig. 1.** *Age and sex distribution of 115 patients with chondrosarcomatous lesions.* □, *male;* ■, *female.*

all the clinical and individual histological parameters included. *p*-Values $< 0.05$ were considered significant; although in the stepwise Cox analysis, the *p*-value for entering parameters was set to 0.05, and for retaining them to 0.10, in order to be able to detect possible borderline significance. The calculations were done on a computer using the 'Stata' statistical software (Stata Corporation, College Station, TX, USA). The inter-observer agreement on the histopathological parameters was evaluated by calculation of overall agreement rates as well as the Kappa statistic.[9] Kappa is 1 if agreement is total, and 0 if it is equal to the expected chance agreement.

### Results

### *Patients*

The age and sex distribution of the 115 patients is shown in Fig. 1. The oldest patient was a 83-year-old man, the youngest a 4-year-old boy. The latter was the only patient with Ollier's disease; four patients had multiple cartilaginous exostoses. Table 2 shows the location of the tumours. Tumour sizes varied between 1.5 and 30 cm (mean, 8.9 cm) in largest diameter, and 54% were judged to be extra-compartmental. Eleven patients were not treated with curative intent, generally because of poor general condition or inoperability (although the size of their tumours did not differ significantly from the rest: $p = 0.36$, *t*-test). In the remaining 104 patients, the margin was judged to be intralesional in 21, marginal in 32, wide in 18 and radical in 32; one was not evaluable. Their 5- and 10-year RFS was 0.62 and 0.58, respectively. A total of 31 tumours recurred locally, one of these with concurrent lung metastases; four recurred with metastases only. After 8 years, no further primary recurrences occurred. Metastases developed with later recurrences in three patients; i.e. metastases occurred altogether in only eight of all 115 patients (7%); one had a grade I tumour that recurred in a dedifferentiated state, four had grade II and three had grade III tumours. Seven of them were men, and seven were extra-compartmental from start, otherwise their data were unremarkable. The OAS of the 104 patients treated with curative intent was 0.75 at 5 years and 0.62 at 10 years (median, 14.7 years), compared with the corresponding rates of only 0.18 and 0 (median, 2.0 years) for the 11 patients who had a biopsy only ($p = 0.0002$, log-rank test).

**Table 2.** *Location of 115 chondromatous tumours (as stratified in the survival analysis)*

| Location | *n* | |
|---|---|---|
| (1) Distal | 19 | (hands 3, feet 4, ulna 1, radius 1, tibia 8, fibula 2) |
| (2) Proximal | 33 | (humerus 8, femur 25) |
| (3) Central | 31 | (pelvis/sacrum 30, spinal column 1) |
| (4) Other | 32 | (costae 12, sternum 5, scapula 12, clavicle 1, maxilla 2) |

### *Univariate analysis*

The results of the log-rank tests for the individual parameters are listed in Table 3. RFS appears to be influenced by tumour size, extra-compartmental growth and surgical margin. Overall survival is not surprisingly correlated to age, but compartment is also prognostically significant. Women have a better prognosis both with regard to RFS and OAS. Surprisingly, conventional grade is not significantly correlated to either RFS or OAS.

### *Multivariate analysis*

The results are listed in Table 4. For RFS, the surgical margin is clearly the most important prognostic factor, as in the univariate analysis, but cellularity and pleomorphism are also statistically significant. Size and number of multinucleated cells approach significance. With regard to OAS, age and surgical margin are the significant clinical factors, while conventional grade together with pleomorphism are the histopathological ones. Note that the coefficient for pleomorphism is negative and the hazard ratio $< 1$; in other words, the more severe the pleomorphism, the better is the prognosis.

Examples of survival curves adjusted for the significant parameters are shown in Figs 2 and 3.

### *Inter-observer variation*

The overall agreement on grade was 0.54 with a Kappa value of 0.32 (0.25–0.39, 95% confidence

**Table 3.** *Results of univariate analysis (p-values, log-rank tests) for 104 patients treated with curative intent*

| No. | Parameter | Stratification | *n* | RFS | OAS |
|---|---|---|---|---|---|
| 1 | Age | ≤30 | 23 | 0.98 | 0.0009 |
| | | 31–60 | 47 | | |
| | | >60 | 34 | | |
| 2 | Sex | Male | 67 | 0.05 | 0.02 |
| | | Female | 37 | | |
| 3 | Site | Distal | 19 | 0.44 | 0.28 |
| | | Proximal | 30 | | |
| | | Central | 25 | | |
| | | Other | 30 | | |
| 4 | Size | ≤5 cm | 26 | 0.02 | 0.54 |
| | | 5–10 cm | 41 | | |
| | | 10–15 cm | 16 | | |
| | | >15 cm | 11 | | |
| | | NA | 10 | | |
| 5 | Compartment | Intra | 47 | 0.01 | 0.01 |
| | | Extra | 54 | | |
| | | NA | 3 | | |
| 6 | Margin | Intralesional | 21 | <0.0001 | 0.09 |
| | | Marginal | 32 | | |
| | | Wide | 18 | | |
| | | Radical | 32 | | |
| | | NA | 1 | | |
| 7 | Invasion | None | 55 | 0.68 | 0.56 |
| | | Present | 49 | | |
| 8 | Cellularity | <300 | 12 | 0.29 | 0.32 |
| | | 300–600 | 32 | | |
| | | 600–900 | 37 | | |
| | | >900 | 23 | | |
| 9 | Pleomorphism | Slight | 36 | 0.11 | 0.17 |
| | | Moderate | 57 | | |
| | | Severe | 11 | | |
| 10 | Multinucleated cells | <10 | 51 | 0.20 | 0.97 |
| | | 10–27 | 40 | | |
| | | >27 | 13 | | |
| 11 | Mitoses | None | 77 | 0.69 | 0.25 |
| | | ≤1 | 18 | | |
| | | >1 | 9 | | |
| 12 | Grade | Benign | 1 | 0.74 | 0.26 |
| | | Borderline | 4 | | |
| | | Grade I | 35 | | |
| | | Grade II | 47 | | |
| | | Grade III | 17 | | |

NA = not available.

For definition of parameters no. 8–11, see Table 1.

limits). Kappa values for the individual grades are shown in Table 5. Best agreement is found for grade III tumours, poorest for the 'borderline' category (the negative Kappa value indicates that this diagnosis by one pathologist is certain to be disputed by the others). The low values for the 'benign' category reflect the bias of sampling, since only suspectedly or obviously malignant tumours were included; the Kappa value will be low if the prevalence of a diagnosis is either very low or very high in the sample.[10] As an experiment, agreement was also calculated after regrouping into only two categories, low (≤I) and high (II + III) grade; this results in an overall agreement of 0.79 with a Kappa value of 0.56 (0.45–0.66), which is in the acceptable range.

Table 6 shows the inter-observer agreement for the individual histopathological variables; the Kappa values are similar to those achieved in grading, only mitotic activity is slightly more reproducible.

### Discussion

Our results stress the overwhelming prognostic importance of the surgical margin (Fig. 2), a finding which is in accordance with the results of other investigators.[2,3,11–13] Extra-compartmental growth

**Table 4.** *Results of stepwise Cox analysis*

| Parameter | Hazard ratio (95% CI) | Coefficient (95% CI) | *p* |
|---|---|---|---|
| RFS | | | |
| Multinucleated | 1.77 (0.93–3.35) | 0.57 (−0.07–1.21) | 0.080 |
| Size | 1.43 (0.97–2.11) | 0.36 (−0.03–0.75) | 0.073 |
| Pleomorphism | 0.35 (0.15–0.82) | −1.04 (−1.88– −0.19) | 0.017 |
| Cellularity | 1.96 (1.15–3.34) | 0.67 (0.14–1.21) | 0.014 |
| Margin | 0.41 (0.27–0.62) | −0.89 (−1.3– −0.47) | 0.000 |
| OAS | | | |
| Age | 1.90 (1.16–3.12) | 0.64 (0.15–1.14) | 0.012 |
| Pleomorphism | 0.38 (0.18–0.77) | −0.97 (−1.69– −0.26) | 0.008 |
| Grade | 2.78 (1.38–5.60) | 1.02 (0.32–1.72) | 0.005 |
| Margin | 0.60 (0.43–0.77) | −0.51 (−0.84– −0.18) | 0.003 |

CI = confidence interval.

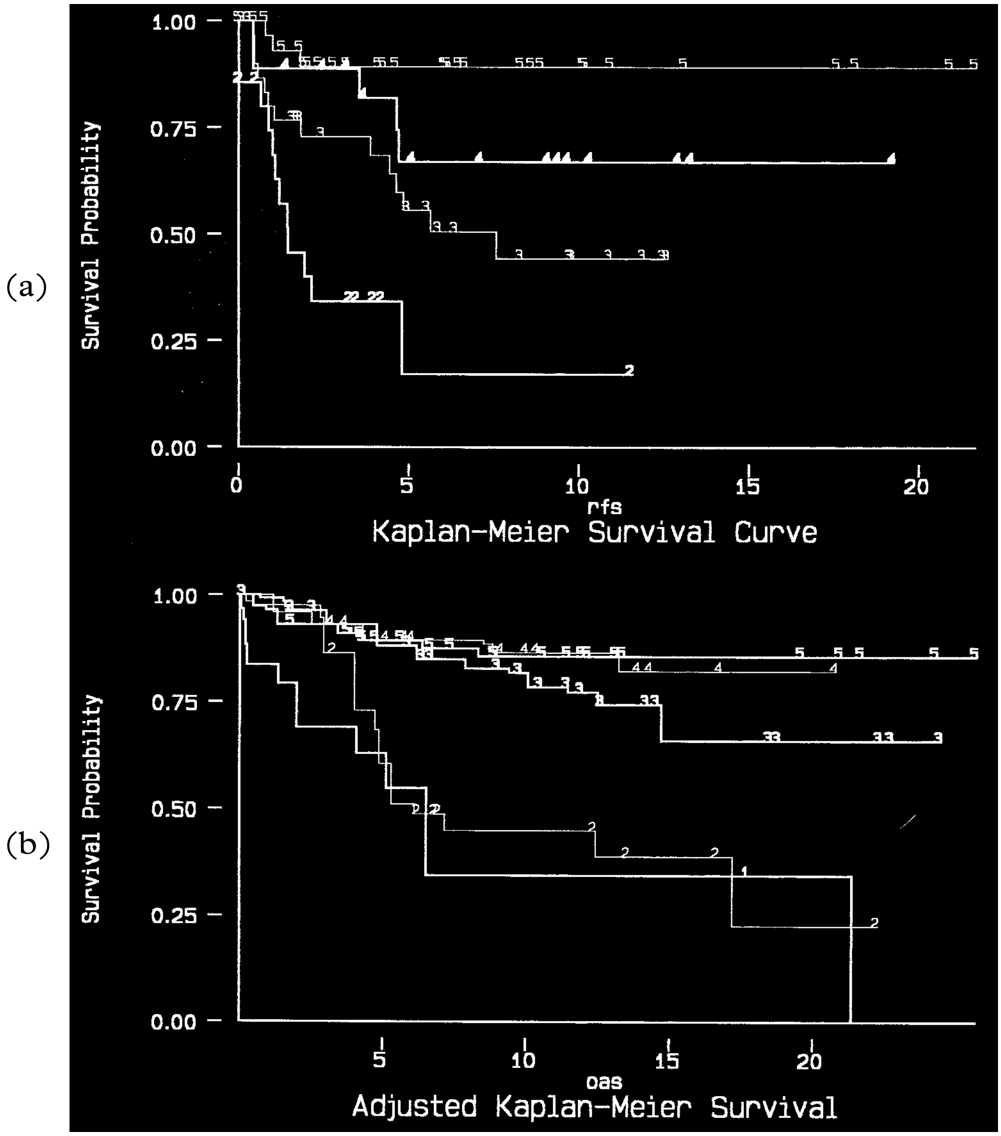

**Fig. 2.** *RFS (a) and OAS (b) as a function of surgical margin: 1, biopsy only; 2, intralesional; 3, marginal; 4, wide; 5, radical. The overall survival curves have been adjusted for age and grade.*

is likewise a bad omen, highly significant in the univariate test, but surprisingly not so in the multivariate analysis–after treatment, the achieved margin is clearly more important than local tumour extension. The site and size of the tumours are only marginally significant; the prognostic infor-

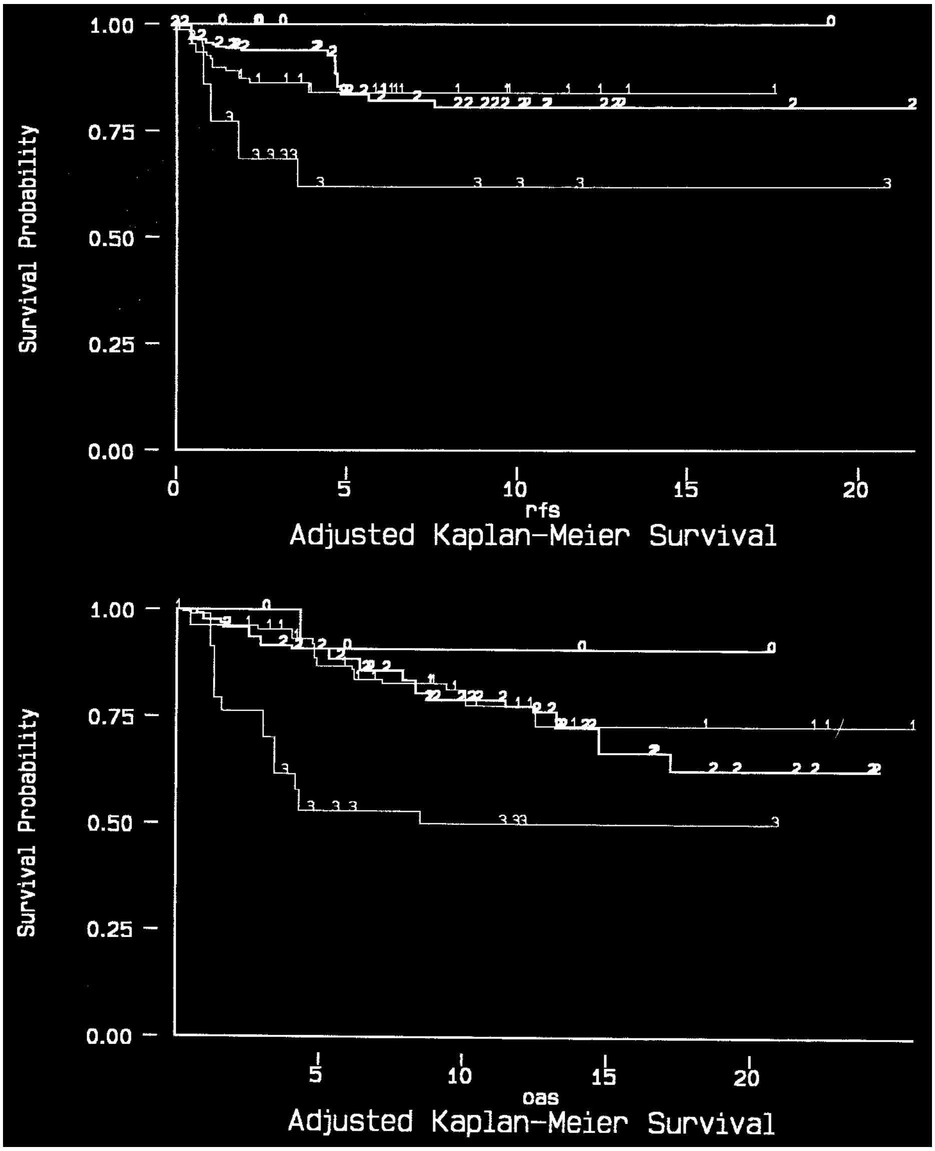

**Fig. 3.** *RFS (a) and OAS (b) as a function of grade: 0-grade < I; 1-grade I; 2-grade II; 3-grade III. The RFS curves have been adjusted for surgical margin and the OAS curves for age and margin.*

mation contained in these two parameters is again surpassed by the importance of the surgical treatment.

The importance of sex is less clear. Like Pritchard *et al.*,[14] we found a better prognosis for women in univariate analysis, but the factor lost its significance when competing with other parameters in the multivariate analysis.

In our material, grading had only moderate prognostic value. No grade I tumours metastasized, but grade was only statistically significant with regard to OAS in the multivariate analysis (Table 4). Only by adjusting for surgical margin and age could the survival curves demonstrate an increasingly poor prognosis with increasing grade (Fig. 3). This is in contrast to the findings of other investigators who in their univariate analyses were able to demonstrate significant correlation between grade and OAS, although RFS appears to be poorly correlated to grade.[2,3,12,14,15] A reason for our finding may be that our grade was a compromise between three observers; another that we may have unduly emphasized nuclear pleomorphism (see later). However, it is worth noting that Burt *et al.*[11] also were unable to demonstrate any influence of grade upon OAS by univariate analysis of their series of 88 chondrosarcomas of the chest wall.

Of the histopathological parameters, invasion and mitotic count were not informative, but the other

**Table 5.** *Inter-observer agreement: Kappa values on grades*

| Grade | Kappa |
|---|---|
| Benign | 0.13 |
| Borderline | −0.07 |
| Grade I | 0.37 |
| Grade II | 0.30 |
| Grade III | 0.48 |

**Table 6.** *Inter-observer agreement on histopathological parameters*

| Parameter | Overall agreement | Kappa (95% CI) |
|---|---|---|
| Cellularity | 0.52 | 0.34 (0.28–0.41) |
| Nuclear pleomorphism | 0.63 | 0.35 (0.27–0.44) |
| Multinucleated cells | 0.63 | 0.36 (0.28–0.44) |
| Mitoses | 0.77 | 0.45 (0.37–0.53) |

CI = confidence interval.

three appear to carry prognostic information, although this only becomes evident in the Cox analysis. Cellularity and nuclear pleomorphism seem to be the most important factors; high cellularity signifies a high grade of malignancy, as would be expected,[16] but it came as a surprise that nuclear pleomorphism was inversely related to the prognosis: the more severe the pleomorphism, the better the prognosis. This feature was constant and could not be explained by covariation with other variables, and it persisted even when the 11 patients with severe pleomorphism were left out from the analysis. It appears to contrast with the finding that nuclear area and DNA content correlate with grade[17,18] and survival.[16] However, a high degree of nuclear pleomorphism (as defined in this study) may not be a reliable indicator of ploidy status, but could also represent a degenerative phenomenon. Thus, nuclear form (as determined by the ratio long axis/short axis) was shown not to correlate with DNA content.[19] The cytogenetics of chondrosarcomas are anyway complex, with no obvious relation between chromosomal pattern and histologic grade,[20] although the number of abnormalities appears to correlate with grade and prognosis.[21]

Our inter-observer agreement on grade was rather poor, a result which confirms the suspicion that conventionally performed grading of chondrosarcomas is highly subjective. The results are similar to those achieved by other investigators in areas where parameters or entities are less clearly defined than generally supposed—for instance, some neurological signs,[22] or subtyping and grading of pulmonary adenocarcinomas.[23] Reducing the number of categories improves agreement, as illustrated by the acceptable values achieved in our material by dividing the lesions solely into low-grade and high-grade lesions. This approach is debatable, however, since it also results in loss of information.[24] In soft tissue sarcomas, inter-observer variation in grading has been reduced to acceptable levels by evaluating histological parameters (differentiation, necrosis and mitotic count) separately.[25] This approach appears to be less useful as regards chondrosarcomas since our results show the inter-observer agreement on the individual histological parameters to be equally poor. On the other hand, cellularity and nuclear pleomorphism or size are features that can be estimated morphometrically[18] and thereby more objectively. Morphometry should therefore be investigated as a means to reducing inter-observer variation to manageable levels.

As mentioned earlier, DNA ploidy has been reported to be a significant independent prognostic factor,[15] but determining this requires special equipment and expertise in interpreting the histograms. Other techniques such as AgNOR counting and immunohistochemical staining for p53 or the proliferation markers PCNA or Ki67 are tricky to perform on chondromatous tissue. They require experience and results have generally been correlated to conventional grade, not clinical outcome.[26–28] However, a recent report by Nawa *et al.*[29] indicates that reactivity with the MIB1 antibody for Ki67 may be a useful independent prognostic marker. Conclusions are difficult to draw, though, because of the rather small numbers of patients investigated in these studies.

In conclusion, the conventional subjective grading of chondrosarcomas is informative but less than optimal, and improvement should be sought for both inter-observer agreement and prognostic information. This will probably need the introduction of new parameters or methods in the grading procedure, but the evaluation of such new methods or factors purported to have prognostic implication will as a minimum require sufficient numbers of patients to allow stratification for age, treatment and grade, best in multivariate analyses. To achieve this, it is necessary to centralize diagnosis and treatment of these rare tumours, and preferably to establish multi-centre cooperation.

### Acknowledgement

This work was supported by a grant from 'Hestehandler af Rønne M. Jensen og hustru Augusta Olivia Jensens mindelegat'.

### References

1 van Oosteroom AT, Dirix LY. Chondrosarcoma and other rare bone sarcomas. *Curr Opin Oncol* 1990; 2:495–9.
2 Evans HL, Ayala AG, Romsdahl MM. Prognostic factors in chondrosarcoma of bone. A clinicopathologic analysis with emphasis on histologic grading. *Cancer* 1977; 40:818–31.

3 Gitelis S, Bertoni F, Picci P, *et al.* Chondrosarcoma of Bone. *J Bone Joint Surg* 1981; 63A:1248–57.
4 Huvos AG. *Bone tumors: diagnosis, treatment, and prognosis*, 2nd edn. Philadelphia: WB Saunders, 1991.
5 Schajowicz F. *Tumors and tumorlike lesions of bone. Pathology, radiology and treatment*, 2nd edn. Berlin: Springer, 1994.
6 Fechner RE, Mills SE. *Tumors of the bones and joints. Atlas of tumor pathology*, 3rd series. Washington DC: Armed Forces Institute of Pathology, 1993.
7 Salisbury JR, Woods CG, Byers PD. *Diseases of bones and joints*. London: Chapman & Hall Medical, 1994.
8 Enneking WF. *Clinical musculoskeletal pathology*, 3rd edn. Gainsville, FL: University of Florida Press, 1990.
9 Svanholm H, Starklint H, Gundersen HJG, *et al.* Reproducibility of histomorphologic diagnoses with special reference to the kappa statistic. *APMIS* 1989; 97:689–98.
10 Gjørup T. The Kappa coefficient and the prevalence of a diagnosis. *Meth Inform Med* 1988; 27:184–6.
11 Burt M, Fulton M, Wessner-Dunlap S, *et al.* Primary bony and cartilaginous sarcomas of chest wall: results of therapy. *Ann Thorac Surg* 1992; 54:226–32.
12 Ozaki T, Lindner N, Hillmann A, *et al.* Influence of intralesional surgery on treatment outcome of chondrosarcoma. *Cancer* 1996; 77:1292–7.
13 Ruark DS, Schlehaider UW, Shah JP. Chondrosarcomas of the head and neck. *World J Surg* 1992; 16:1010–16.
14 Pritchard DJ, Lunke RJ, Taylor WF, *et al.* Chondrosarcoma: a clinicopathologic and statistical analysis. *Cancer* 1980; 45:149–57.
15 Kreicbergs A, Boquist L, Borssén B, *et al.* Prognostic factors in chondrosarcoma. A comparative study of cellular DNA content and clinicopathologic features. *Cancer* 1982; 50:577–83.
16 Kreicbergs A, Slezak E, Söderberg G. The prognostic significance of different histomorphologic features in chondrosarcoma. *Virchows Arch A Pathol Anat Histol* 1981; 390:1–10.
17 Zeppa P, Zabatta A, Marino G, *et al.* A morphometric approach to the grading of chondroid tumours on fine-needle smears. *Pathol Res Pract* 1989; 185:760–3.
18 Ishida T, Kikuchi F, Machinami R. Histological grading and morphometric analysis of cartilaginous tumours. *Virchows Arch A Pathol Anat* 1991; 418:149–55.
19 Kreicbergs A, Zetterberg A. Cytophotometric DNA measurements of chondrosarcoma. *Analyt Quant Cytol* 1980; 2:84–92.
20 Dijkhuizen T, van den Berg E, Molenaar WM, *et al.* Cytogenetics as a tool in the histologic subclassification of chondrosarcomas. *Cancer Genet Cytogenet* 1994; 76:100–5.
21 Bridge JA, Bhatia PS, Anderson JR, *et al.* Biologic and clinical significance of cytogenetic and molecular cytogenetic abnormalities in benign and malignant cartilaginous lesions. *Cancer Genet Cytogenet* 1993; 69:79–90.
22 Hansen M, Sindrup SH, Christensen PB, *et al.* Interobserver variation in the evaluation of neurological signs: observer dependent factors. *Acta Neurol Scand* 1994; 90:145–9.
23 Sørensen JB, Hirsch FR, Gazdar A, *et al.* Interobserver variability in histopathologic subtyping and grading of pulmonary adenocarcinoma. *Cancer* 1993; 71:2971–6.
24 Morris JA. Information and observer disagreement in histopathology. *Histopathology* 1994; 25:123–8.
25 Coindre JM, Trojani M, Contesso G, *et al.* Reproducibility of a histopathologic grading system for adult soft tissue sarcoma. *Cancer* 1986; 58:306–9.
26 Ohno T, Tanaka T, Takeuchi S, *et al.* Silver-stained nucleolar organizer proteins in chondrosarcoma. *Virchows Archiv B Cell Pathol* 1991; 60:207–11.
27 Coughlan B, Feliz A, Ishida T, *et al.* p53 expression and DNA ploidy of cartilage lesions. *Hum Pathol* 1995; 26:620–4.
28 Hasegawa T, Seki K, Yang P, *et al.* Differentiation and proliferative activity in benign and malignant cartilage tumors of bone. *Hum Pathol* 1995; 26:838–45.
29 Nawa G, Ueda T, Mori S, *et al.* Prognostic significance of Ki67 (MIB1) proliferation index and p53 over-expression in chondrosarcomas. *Int J Cancer* 1996; 69:86–91.
